# Real-time characterization of risks of death associated with the Middle East respiratory syndrome (MERS) in the Republic of Korea, 2015

**DOI:** 10.1186/s12916-015-0468-3

**Published:** 2015-09-30

**Authors:** Kenji Mizumoto, Akira Endo, Gerardo Chowell, Yuichiro Miyamatsu, Masaya Saitoh, Hiroshi Nishiura

**Affiliations:** Graduate School of Arts and Sciences, The University of Tokyo, 3-8-1 Komaba, Meguro-ku, Tokyo 1538902 Japan; Graduate School of Medicine, The University of Tokyo, 7-3-1 Hongo, Bunkyo-ku, Tokyo 1130033 Japan; School of Public Health, Georgia State University, Atlanta, Georgia USA; Division of International Epidemiology and Population Studies, Fogarty International Center, National Institutes of Health, Bethesda, Maryland USA; CREST, Japan Science and Technology Agency, Honcho 4-1-8, Kawaguchi, Saitama 332-0012 Japan; The Institute of Statistical Mathematics, Tachikawa, Tokyo Japan

**Keywords:** Case fatality ratio, Fatal outcome, Middle East respiratory syndrome, Risk factors, Statistical model, South Korea

## Abstract

**Background:**

An outbreak of the Middle East respiratory syndrome (MERS), comprising 185 cases linked to healthcare facilities, occurred in the Republic of Korea from May to July 2015. Owing to the nosocomial nature of the outbreak, it is particularly important to gain a better understanding of the epidemiological determinants characterizing the risk of MERS death in order to predict the heterogeneous risk of death in medical settings.

**Methods:**

We have devised a novel statistical model that identifies the risk of MERS death during the outbreak in real time. While accounting for the time delay from illness onset to death, risk factors for death were identified using a linear predictor tied to a logit model. We employ this approach to (1) quantify the risks of death and (2) characterize the temporal evolution of the case fatality ratio (CFR) as case ascertainment greatly improved during the course of the outbreak.

**Results:**

Senior persons aged 60 years or over were found to be 9.3 times (95 % confidence interval (CI), 5.3–16.9) more likely to die compared to younger MERS cases. Patients under treatment were at a 7.8-fold (95 % CI, 4.0–16.7) significantly higher risk of death compared to other MERS cases. The CFR among patients aged 60 years or older under treatment was estimated at 48.2 % (95 % CI, 35.2–61.3) as of July 31, 2015, while the CFR among other cases was estimated to lie below 15 %. From June 6, 2015, onwards, the CFR declined 0.3-fold (95 % CI, 0.1–1.1) compared to the earlier epidemic period, which may perhaps reflect enhanced case ascertainment following major contact tracing efforts.

**Conclusions:**

The risk of MERS death was significantly associated with older age as well as treatment for underlying diseases after explicitly adjusting for the delay between illness onset and death. Because MERS outbreaks are greatly amplified in the healthcare setting, enhanced infection control practices in medical facilities should strive to shield risk groups from MERS exposure.

## Background

When a novel infectious disease emerges, it is vital to assess the potential global public health impact, i.e. the potential of a pathogen to cause a devastating pandemic [1]. The public health impact is mainly characterized by the transmission potential and virulence, the latter of which is usually measured by the case fatality ratio (CFR), namely the proportion of deaths among cases [2]. Due to the importance of generating accurate estimates of the CFR in real time as an outbreak progresses and public health interventions are implemented, a number of epidemiological methods have been proposed, including those that account for the time delay from illness onset to death [3–5] and those that correct for ascertainment bias through the combination of several different observations by employing a Bayesian evidence synthesis method [6]. Other methods have developed a slightly different CFR concept, e.g. the so-called “infection fatality risk”, the ratio of excess mortality to the serologically determined infected fraction of the population [7]. The CFR is an important epidemiological quantity to estimate in real time during outbreaks, and the recent outbreak of the Middle East respiratory syndrome (MERS) in the Republic of Korea in 2015 has not been an exception [8]. Besides the MERS outbreak in the Republic of Korea, earlier studies of MERS in the Middle East have consistently indicated that the CFR for MERS has been about 40 % [9] and could be as low as 20 % for estimates that only include secondary cases [10].

Despite the availability of useful methods to estimate the CFR for MERS, it is crucial to identify risk factors associated with MERS death, as the risk of death may vary significantly with age, occupation, and underlying comorbidities [11]. During the epidemic of severe acute respiratory syndrome (SARS), a real-time analysis found that confirmed or probable cases aged 60 years or older were at greater risk of death than younger cases [12]. Moreover, infections with SARS-associated coronavirus with comorbidities were found to be 1.7 times more likely to die than those without comorbidities [13]. Due to similarities in clinical characteristics between MERS and SARS patients as both coronaviruses are closely related [9], we can expect that the risk of MERS death can also be similarly characterized by a set of underlying epidemiological features of the cases. While the methods for identifying the risk of death in real time during the SARS epidemic have relied on survival analysis techniques, including non-parametric Kaplan-Meier-like methods [12, 14], there is a need to develop a simple yet tractable method which is inspired on the adjustment of censoring for CFR [4, 5] with particular application to small outbreak sizes such as the MERS outbreak in the Republic of Korea. Such a modelling framework should adjust for case ascertainment, which is widely recognized as a key issue that arises during emerging disease epidemics, including the H1N1 pandemic in 2009 [2, 7, 8]. During the MERS outbreak in the Republic of Korea, an extensive contact tracing effort was made by public health authorities soon after the first few cases were reported, with all cases being confirmed by laboratory testing irrespective of clinical signs and symptoms. As in any outbreak, it is likely that the ascertainment rate was substantially improved as the outbreak progressed.

The present study aims to statistically identify risk factors associated with MERS death in the Republic of Korea using a simple but novel statistical modelling approach. We also use our approach to assess the time dependent variation in the ascertainment of cases.

## Methods

### Epidemiological data

The present study employs published secondary data of the confirmed MERS cases arising from the outbreak in the Republic of Korea [15–17]. As of July 31, 2015, a total of 185 cases have been diagnosed (excluding one case diagnosed and recovered in China) including 36 deceased cases. During the course of this outbreak, a detailed line list of cases has been made publicly available [15]. Data on individual cases include the (1) dates of illness onset, (2) age-group, (3) gender, and (4) background health status either as outpatient or inpatient of a specific healthcare facility. Since the dates of illness onset among those recently reported were not yet available, we relied on the dates of confirmatory diagnosis as an alternative. We believe it is reasonable to approximate the date of illness onset by the date of confirmed diagnosis in this setting, because from the midst of the outbreak, all suspected contacts have been regularly monitored and have been subjected to laboratory testing regardless of clinical signs and symptoms. We limited ourselves to handle the abovementioned covariates (2) to (4) only, not each specific individual comorbidity, because it is not clear if the presence of all common underlying comorbidities were routinely evaluated and consistently documented in the case list; the identification of most closely associated specific comorbidities may be the subject of future clinical studies.

### Statistical model

Herein, we develop an estimation model composed of a mixture of a survival model and logistic regression model. Let *S*(*τ*) and *p* be the survival probability at time *τ* since illness onset and the CFR, respectively. The relationship of the two is described as

i $$ p=1-S\left(\infty \right), $$

where *S*(*τ*) is given by

ii $$ S\left(\tau; p\right)=1-p{\displaystyle \underset{0}{\overset{\tau }{\int }}f(x)}dx, $$

where *f*(*τ*) is the conditional probability density function of the time from illness onset to death given fatal outcome [4, 18]. In the majority of the following analyses, *f*(*τ*) was assumed as known and based on the data from first 10 reported cases who eventually resulted in death in South Korea, using the moment-based estimates of the mean (13.2 days) and standard deviation (7.1 days). Nevertheless, we also examined the heterogeneous risk of death by jointly quantifying heterogeneous risk factors and parameters of *f*(*τ*), verifying that the estimates of the risk of death in the joint estimation do not greatly differ from those we obtained using the assumed known *f*(*τ*).

Since we aim to identify risk factors (or explanatory variables) of *p*, the notation *p* is set to be changeable by individual *i*, i.e. *p*_i_. Such individual variation is modelled using the logit model:

iii $$ \ln \left(\frac{p_i}{1-{p}_i}\right)={a}_0+{\displaystyle \sum_{k=1}^N{a}_k{x}_{k,i}}, $$

where *a*_0_ is the intercept, *a*_k_ the coefficient of variable *k*, *x*_k,i_ the *k*-th variable of individual *i* of the linear predictor, and *N* the total number of independent variables. Let *A* and *B* represent the groups of cases who have survived and died by the most recent calendar time *t*_m_, the likelihood function to parameterize the linear predictor in (iii) is

iv $$ L\left(\mathbf{a};\boldsymbol{\upalpha}, \boldsymbol{\upbeta}, {t}_m\right)=\prod_{i\in A}S\left({t}_m-{\alpha}_i;{p}_i\right)\prod_{i\in B}\left[{p}_if\left({\beta}_i-{\alpha}_i\right)\right], $$

where *α*_i_ and *β*_i_ represent the observed dates of illness onset and death of an individual *i*, respectively, with coefficient vector **a** = (*a*_0_, *a*_1_, ⋯, *a*_*N*_).

### Estimation settings

In our analysis of the Korean MERS data in real time, univariate analyses using the abovementioned logistic regression were conducted to detect any variable associated with the risk of death. Explanatory dichotomous variables include age-group (below or above 60 years old), gender, and patients under treatment. Patients under treatment include both outpatients and inpatients, while non-patient cases represent all other cases including healthcare workers, visitors, and so on. The univariate test was achieved by estimating the CFR at the most recent time *t*_m_ for each subgroup using the solution of *p*_i_ obtained from (iii) and calculating the difference of CFR between the estimates. After identifying variables that were significantly associated with MERS death from univariate analyses, a multivariate version of the model (iii) was run to adjust confounding factors and identify epidemiological factors that were significantly associated with death.

Subsequently, we used exactly the same model as (iii) to test if there was any time-dependent change in the risk of death, perhaps due to increased ascertainment involving diagnoses of a substantial number of mild and asymptomatic cases. The time dependence in the CFR was modelled by introducing a parameter *δ*, a constant factor multiplied to the original logit model, i.e.

v $$ {p}_i=\frac{\delta \exp \left({a}_0+{\displaystyle \sum_{k=1}^N{a}_k{x}_{k,i}}\right)}{1+ \exp \left({a}_0+{\displaystyle \sum_{k=1}^N{a}_k{x}_{k,i}}\right)}, $$

where *δ* was dealt with as

vi $$ \delta =\left\{\begin{array}{l}1,\kern2.75em \mathrm{f}\mathrm{o}\mathrm{r}\ t<{t}_0\ \\ {}\varepsilon, \kern2.75em \mathrm{f}\mathrm{o}\mathrm{r}\ {t}_0\le t\end{array}\right., $$

where *t*_0_ represents the first day on which the ascertainment rate increased while *ε* scales the extent of ascertainment (where *ε* is expected to be less than 1); *t*_0_ was objectively sought by comparing the Akaike Information Criterion (AIC) among models with different *t*_0_.

When the joint estimation was conducted, we estimated not only coefficients of the linear predictor in the likelihood *L* but also parameters for *f*(*τ*), i.e. mean and standard deviation of the gamma distribution. Parameters were estimated using the maximum likelihood method (i.e. by minimizing negative logarithm of (iv)). The 95 % confidence intervals (CI) were derived from the profile likelihood. Different models were compared using the AIC.

### Ethical considerations

The present study reanalyzed the publicly available secondary data from the Korean Government and WHO which collected the notification data with ethical approval and written consent from patients, and adhering to the International Health Regulations. The secondary data were de-identified by these organizations in advance of our access. As such, the datasets employed in our study have been deemed exempted from the ethical approval.

### Availability of supporting data

The present study fully rests on published data, and essential components of the data consisting of dates of illness onset and death are downloadable from the WHO website [15].

## Results

### Risk factors associated with MERS death

Table 1 shows the results of univariate analyses. Cases among senior persons aged 60 years or over (*P* <0.01) and patients under treatment (*P* <0.01) were significantly associated with MERS death: senior persons appeared to be 9.3-fold (95 % CI, 5.3–16.9) more likely to die compared to younger cases and patients under treatment were 7.8-fold (95 % CI, 4.0–16.7) more likely to be fatal than others. Gender was not found to be a significant predictor of death (*P* = 0.57).

**Table 1:** Univariate association with the risk of death from Middle East respiratory syndrome (MERS) (n = 185)

Variables	Univariate odds ratio (95 % confidence intervals)	*P* value
Age (≥60 years old)	9.3 (5.3–16.9)	<0.01
Sex (female to male)	1.3 (0.8–2.1)	0.57
Patients under treatment	7.8 (4.0–16.7)	<0.01

Univariate odds ratio was derived from the logit model with time-delay from illness onset to death, while *P* values were derived from Fisher’s exact test

**Fig. 1:**
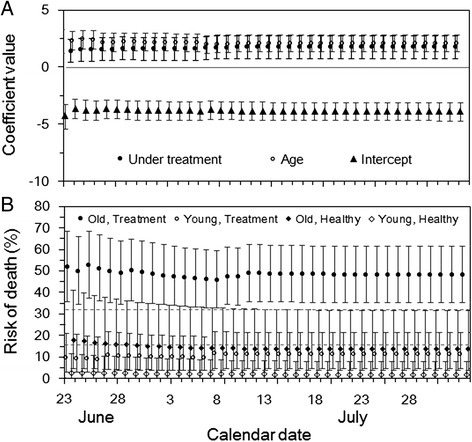
Estimated case fatality ratios of Middle East respiratory syndrome (MERS) by subgroups in the Republic of Korea, 2015. **a** Estimated value of regression coefficient as a function of calendar time. The horizontal straight grey line represents zero value. **b** Estimated case fatality ratio (CFR) by subgroup as a function of calendar time. The horizontal dashed grey lines correspond to the published value of the CFR (i.e., 20 % and 40 %) [9, 10]. In both panels, the horizontal axis represents the date at which the estimation was implemented. Upper and lower 95 % confidence intervals (CI) for each parameter derived from the profile likelihood are indicated by the whiskers

Table 2 summarizes the results derived from our multivariate model analyses and includes the smallest AIC value. Cases among senior persons and patients under treatment were included in the final model. Figure 1a indicates that the estimates of coefficients remained stable over time, whereas Figure 1b illustrates the estimated CFR by subgroups. As of July 31, 2015, the CFR among patients aged 60 years or older under treatment was estimated at 48.2 % (95 % CI, 35.2–61.3). The CFRs in other subgroups were low and estimated as follows: 1.8 % in the lowest risk group (age <60 years without any treatment), 13.6 % among those aged 60 years or older without any treatment, and 11.1 % among those aged younger than 60 years old under treatment.

**Table 2:** Predicted risk factors of death from Middle East respiratory syndrome (MERS) death using a real-time multivariate model

Variables	Coefficient ^a^ (95 % confidence interval)	Adjusted odds ratio (95 % confidence interval)
Intercept	−3.9 (−4.7 to −3.1)	0.02 (0.01–0.04)
Age (≥60 years old)	2.0 (1.4–2.6)	7.6 (4.2–14.0)
Patients under treatment	1.8 (1.1–2.6)	5.9 (2.9–12.8)

Negative log likelihood = 199.7; Dependent nominal variable = death

^a^ Coefficient of the linear predictor

### Time dependent risk of MERS death

Using the time-dependent logit model, we quantified the impact of improved case ascertainment. Importantly, the date at which the risk of death most likely declined was estimated to be June 6, 2015, as determined by the minimum AIC value (401.9) with models (v) and (vi). *ε* was estimated to be 0.3 (95 % CI, 0.1–1.1), indicating that the case fatality ratio sharply declined by 70 % from June 6, 2015, onwards. Although the estimated relative change in the case fatality ratio was not significantly below 1, it should be noted that the AIC value of the model with time-dependent decline in the CFR was smaller than the alternative model without time-dependent decline in the risk of death (AIC = 405.3).

### Estimated time from illness onset to death

**Fig. 2:**
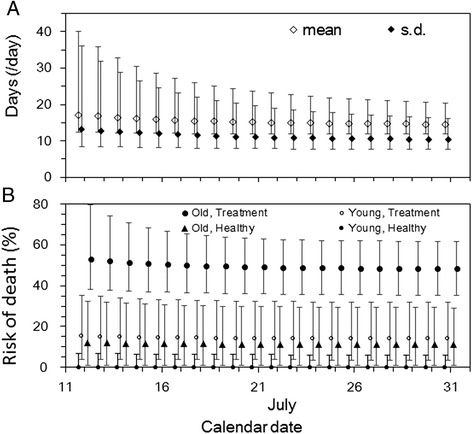
Joint estimation of the time from illness onset to death and type-specific case fatality ratio of Middle East respiratory syndrome (MERS) in the Republic of Korea, 2015. **a** Mean and standard deviation of the time from illness onset to death. **b** Estimated case fatality ratio (CFR) by subgroup as a function of calendar time. In both panels, the horizontal axis represents the date at which the estimation was implemented. Upper and lower 95 % confidence intervals (CI) for each parameter derived from the profile likelihood are indicated by the whiskers

Jointly quantifying the time from illness onset to death, the mean and standard deviation of the delay were estimated at 14.6 (95 % CI, 11.5–19.5) days and 10.4 (95 % CI, 7.6–16.2) days, respectively. The fixed mean and standard deviation using first 10 deceased cases were not significantly deviated from these estimates. Accordingly, the estimated CFR by different types of individuals were also not significantly different from those described above (Fig. 2). As of July 31, 2015, the CFR among patients aged 60 years or older under treatment was estimated at 48.4 % (95 % CI, 35.4–61.6). The CFRs in other subgroups were 1.8 % in the lowest risk group (age <60 years without any treatment), 13.7 % among those aged 60 years or older without any treatment, and 11.2 % among those aged younger than 60 years old under treatment. The AIC value of the most up-to-date joint model was 406.7, i.e. a value greater than that obtained using the fixed mean and the standard deviation. It should be noted that the joint estimation model resulted in successful convergence only on and after July 12, 2015, i.e. 10 days since illness onset of the latest case on July 2, 2015.

## Discussion

The present study has successfully devised a novel hybrid statistical model to characterize the heterogeneous risk of death associated with MERS in real time in the context of the most recent MERS outbreak in the Republic of Korea from May to July 2015. Our method, relying on the logit model, enabled us not only to identify epidemiological determinants of MERS death but also to examine the time-dependent variation in CFR due to an increased case ascertainment rate influenced by extensive contact tracing efforts. We have shown that cases among senior persons under treatment were at particularly high risk of MERS death. In addition, we have found that the CFR most likely declined since June 6, 2015, by about 70 %.

We found marked similarities with SARS as the CFR for MERS among patients aged 60 years or older under treatment was estimated as high as 48.2 % (95 % CI, 35.2–61.3), while that for younger healthy individuals was less than 15 % [12, 13]. Our CFR estimate for senior persons was consistent with an overall CFR estimate of 40 % from prior studies [9]. Moreover, it can also explain a decline in the CFR estimate among secondary cases in the Middle East at 20 % [10, 19]: most secondary transmissions were among healthcare workers and visitors without underlying comorbidities. Because MERS outbreaks tend to be amplified in healthcare settings comprised by a significant fraction of vulnerable populations (e.g. older populations with underlying comorbidities), an important caveat derived from our analysis is that it is critical to enhance and maintain infection control measures in healthcare settings not only to avoid secondary transmissions but also mitigate the mortality burden. Countermeasures that minimize infections among patients in healthcare settings, e.g. restricting the number of visits to different hospitals per case, should be taken into consideration.

It should be noted that the present study is not the first to utilize statistical methods to estimate the heterogeneous risks of death in real time. Specifically, prior studies on SARS have employed non-parametric survival analysis during the course of the epidemic [3, 12] and a method for estimating relative case fatality was proposed elsewhere [20]. However, compared to earlier models, the proposed model was shown to be advantageous in two aspects. First, our approach allowed us to pinpoint a sharp decline in the CFR over time by using only a single multiplicative parameter. Indeed, the reason behind a decline in CFR by 70 % since June 6, 2015, is consistent with an increased case ascertainment due to extensive contact tracing efforts during the outbreak which has inherently increased the diagnosis of very mild cases. If we aim to estimate the risk of death among all infected individuals [7], the corresponding estimate is likely lower than what we presented here. Second, as we employed the logistic type model, our method is able to handle very small sample sizes for each subgroup of cases. By using the logit model, our approach was able to successfully identify risk factors from very small samples (i.e. 185 cases and 36 deaths) for the MERS outbreak in the Republic of Korea.

In addition to characterizing the heterogeneous and time dependent risks of MERS death, we have also estimated the time from illness onset to death jointly with other parameters, confirming that the risk estimates of each group were consistent with those derived from a model with fixed mean and standard deviation of the time from illness onset to death. The goodness-of-fit assessed by AIC indicated that joint estimation did not contribute to improve model fit to the data in a comparative sense, but as practiced in the present study, the joint estimation procedure can help verify the CFR estimates of each risk group using the known distribution of time from illness onset to death.

Two limitations have to be noted. First, our exercise involved only a limited number of covariates as potential determinants of MERS death. The publicly available case list sometimes included descriptions regarding specific comorbidities, but we were not able to ensure that all common comorbidities were systematically and consistently documented in the list. Second, we had to reach a compromise with some data gaps. In particular, whenever the date of illness onset was unavailable, the date of confirmatory diagnosis was used as a proxy. However, considering that Korean public health investigators have successfully conducted major contact tracing efforts and tested all suspected cases, the difference between these two time variables appeared to have been small (e.g. among cases who developed illness in the latter half of June 2015, the median time elapsed from illness onset to diagnosis was only 2 days). The same issue applies to the interpretation of the date of death. In contrast to the substantial differences between the actual and reported date of death for cases of the Ebola virus disease outbreaks in West Africa, the close monitoring of MERS cases in South Korea enabled us to analyze the actual date of death in real time.

Our study was aimed to identify key determinants of MERS death using a simple framework in real time, and future studies could aim to carry out a more thorough analysis of the final dataset that could include more information. While there are certainly further opportunities to improve our methods and conduct further similar studies in other settings, our statistical approach has allowed us to successfully identify patients aged 60 years or older under treatment at particularly high-risk of MERS death in real time. Our model also helped us capture the impact of increased ascertainment in Korea as reflected in our estimate of the CFR from the midst of the outbreak. Considering that MERS tends to be amplified in healthcare settings [21], the need to enhance and maintain strict infection control measures at hospitals to avoid secondary transmissions, particularly among high risk groups, cannot be overemphasized.

## Conclusion

The risk of MERS death was significantly associated with older age as well as treatment for underlying diseases after explicitly adjusting for the delay between illness onset and death. Because MERS outbreaks are greatly amplified in the healthcare setting, enhanced infection control practices in medical facilities should strive to shield risk groups from MERS exposure. The proposed model can be used for other outbreaks of emerging infectious diseases including those involving small number of cases and time-dependent variation in the ascertainment rate.

## Abbreviations

AIC: Akaike Information Criterion
CFR: Case fatality ratio
CI: Confidence interval
MERS: Middle East respiratory syndrome
SARS: Severe acute respiratory syndrome
